# ANTHROPOMETRY AND CLUSTERED CARDIOMETABOLIC RISK FACTORS IN YOUNG PEOPLE: A SYSTEMATIC REVIEW

**DOI:** 10.1590/1984-0462/;2017;35;3;00013

**Published:** 2017-07-31

**Authors:** Teresa Maria Bianchini de Quadros, Alex Pinheiro Gordia, Luciana Rodrigues Silva

**Affiliations:** a Universidade Federal do Recôncavo da Bahia, Amargosa, BA, Brasil.; b Faculdade de Medicina, Universidade Federal da Bahia, Salvador, BA, Brasil.

**Keywords:** Child, Adolescent, Overweight, Obesity, Cardiovascular diseases, Body mass index

## Abstract

**Objective:**

To conduct a systematic review of the literature on the ability of anthropometric indicators to predict clustered cardiometabolic risk factors (CMRF) in children and adolescents.

**Data source:**

Studies published from June 1^st^, 2011 to May 31^st^, 2016 in the PubMed, SciELO and LILACS databases were analyzed. The research was based on keywords derived from the terms “anthropometric indicators” AND “cardiometabolic risk factors”. Observational studies on the ability of anthropometric indicators as predictors of clustered CMRF in children and adolescents in Portuguese, English and Spanish languages were included. Studies with a specific group of obese patients or with other diseases were not included.

**Data synthesis:**

Of the 2,755 articles retrieved, 31 were selected for systematic review. Twenty-eight studies analyzed body mass index (BMI) as a predictor of clustered CMRF. Only 3 of the 25 cross-sectional studies found no association between anthropometric indicators and clustered CMRF. The results of six studies that compared the predictive ability of different anthropometric measures for clustered CMRF were divergent, and it was not possible to define a single indicator as the best predictor of clustered CMRF. Only six articles were cohort studies, and the findings suggested that changes in adiposity during childhood predict alterations in the clustered CMRF in adolescence.

**Conclusions:**

BMI, waist circumference and waist-to-height ratio were predictors of clustered CMRF in childhood and adolescence and exhibited a similar predictive ability for these outcomes. These findings suggest anthropometric indicators as an interesting screening tool of clustered CMRF at early ages.

## RESUMO

**Objetivo:**

Revisar sistematicamente a literatura sobre a habilidade de indicadores antropométricos para predizer fatores de risco cardiometabólico (FRC) agrupados em crianças e adolescentes.

**Fonte de dados:**

Foram analisados estudos publicados de 1º de junho de 2011 até 31 de maio de 2016 nas bases PubMed, SciELO e LILACS. A pesquisa baseou-se em palavras-chave derivadas dos termos “indicadores antropométricos” AND “fatores de risco cardiometabólico”. Foram incluídos estudos observacionais sobre a habilidade de indicadores antropométricos como preditores de FRC agrupados em crianças e adolescentes, nos idiomas português, inglês e espanhol. Não foram incluídos estudos com grupo específico de pacientes com obesidade ou outras doenças.

**Síntese dos dados:**

Dos 2.755 registros encontrados, 31 estudos foram selecionados para revisão sistemática. Vinte e oito estudos analisaram a habilidade do índice de massa corporal (IMC) como preditor de FRC agrupados. Dos 25 estudos transversais, apenas em 3 não foi observada associação entre indicadores antropométricos e FRC agrupados. Os resultados dos seis estudos que compararam a habilidade de diferentes medidas antropométricas como preditoras de FRC agrupados foram divergentes, não sendo possível definir um único indicador como melhor preditor de FRC agrupados. Apenas seis estudos eram de coorte, e os achados sugeriram que mudanças na adiposidade na infância predizem alterações nos FRC agrupados na adolescência.

**Conclusões:**

O IMC, o perímetro da cintura e a relação cintura-estatura foram preditores de FRC agrupados na infância e na adolescência e apresentaram habilidade similar para predizer esses desfechos. Esses achados sugerem que indicadores antropométricos podem representar uma interessante ferramenta para triagem epidemiológica de FRC agrupados em idades precoces.

## INTRODUCTION

Body mass index (BMI) has been used for decades to assess overweight and obesity.^1^ Likewise, the waist perimeter (WP) is used to assess central adiposity, and the waist-to-height ratio (WHtR) came from the need to correct the WP measure due to the growth of children and adolescents.^2^ ^,^ ^3^ With the increasing incidence of cardiometabolic risk factors (CMRF) in the pediatric population, low-cost, non-invasive, easy-to-measure and possible large-scale evaluation methods have been exhaustively studied by the scientific community.^4^ ^,^ ^5^ ^,^ ^6^ Therefore, anthropometric measurements are suggested as CMRF predictors in childhood and adolescence.^4^ ^,^ ^5^ ^,^ ^6^

According to the systematic review conducted with articles published until 2014, with the objective of verifying the association between abdominal obesity and CMRF in children and adolescents, regardless of the definition used for abdominal obesity and the methods used for anthropometric measurements, central fat deposition in children and adolescents increases the risk of CMRF.^4^ Two other important systematic reviews were published in 2010.^5^ ^,^ ^6^ Browning et al.^5^ sistematically reviewed studies that support WHtR as a predictor of CMRF in adults and children, besides reporting relations between WHtR, BMI or WP, or both. Of the revised studies, 13 were conducted with children and adolescents - all cross-sectional analyses. The findings of the review showed that WHtR and WP were more strongly associated with isolated CMRF than BMI.^5^ A systematic review conducted by Reilly et al.,^6^ who analyzed studies comparing the accuracy (area under the curve - AUC) of BMI and WP to predict CMRF, showed that the AUC of both measurements in the CMRF diagnosis were similar.^6^

Subcutaneous fat accumulation measured by skinfolds (SF) has also proven to be a good predictor of CMRF in adolescents.^7^ However, none of the aforementioned systematic reviews included this measurement in the search. Nonetheless, according to the synthesis of these reviews, it is possible to point out some gaps. In the reviews by Kelishadi et al.^4^ and Browning et al.,^5^ the authors did not verify any differences between anthropometric measures, and did not focus the review on clustered CMRF. In the review by Reilly et al.,^6^ the authors compared the ability of only two anthropometric measurements, and only three studies presented two or more clustered CMRF as outcomes. According to the *Bogalusa Heart Study,* adverse levels of clustered CMRF tend to coexist in the same individual from childhood to adulthood.^8^ The identification of simple methods enabling the epidemiological screening of clustered CMRF in the pediatric population may represent a useful strategy to reduce the incidence of cardiometabolic conditions throughout life. In this sense, this systematic review aimed to verify the ability of anthropometric indicators to predict clustered CMRF in children and adolescents.

## METHOD

This study is a systematic review conducted in accordance with the Preferred Reported Items for Systematic Reviews and Meta-Analyses (PRISMA) methodology.^9^ In addition, the Cochrane manual for systematic reviews^10^ was consulted during the development of the study. The study protocol was not registered in the International Prospective Register of Systematic Reviews (PROSPERO) databases.

Studies published from June 1^st^, 2011, to May 31^st^, 2016 in PubMed, SciELO and LILACS databases were evaluated. The search strategy used in PubMed is demonstrated as follows, and the same research terms were used in the other databases: (“body mass index”[All Fields] OR “BMI”[All Fields] OR “waist circumference”[All Fields] OR “WC”[All Fields] OR “waist perimeter”[All Fields] OR “skinfolds”[All Fields] OR “skinfold thickness”[All Fields] OR “Waist-Height Ratio”[All Fields] OR “WHtR”[All Fields] OR “waist to height ratio”[All Fields]) AND (“cardiovascular risk factors”[All Fields] OR “cardiovascular disorders”[All Fields] OR “cardiovascular risk”[All Fields] OR “metabolic syndrome”[All Fields] OR “metabolic risk”[All Fields] OR “metabolic risk factors”[All Fields] OR “metabolic disorders”[All Fields] OR “cardiometabolic risk”[All Fields] OR “cardiometabolic risk factors”[All Fields] OR “cardiometabolic disorders”[All Fields]) NOT (review[Publication Type] OR randomized controlled trial[Publication Type] OR controlled clinical trial[Publication Type]) AND ((“2011/06/01”[PDAT]: “2016/05/31”[PDAT]) AND “humans”[MeSH Terms] AND (“child”[MeSH Terms:noexp] OR “adolescent”[MeSH Terms])).

In this study, clustered CMRF were defined as the simultaneous presence of two or more of the following conditions: high blood pressure, hyperglycemia, sensitivity to insulin, resistance to insulin, hypertriglyceridemia, high total cholesterol, high LDL-cholesterol, high VLDL-cholesterol and low HDL-cholesterol.

Bibliographic search was conducted by two independent researchers, who initially screened the titles and abstracts of the articles, and the relevant articles were selected to be read in full. Duplicated articles were removed.

To be included in the systematic review, the studies had to meet the following criteria:

1. To investigate the ability of anthropometric indicators as predictors of clustered CMRF.
2. To report data of children and adolescents (aged between 6 and 17.9 years, or part of this age group, or mean age in this interval).
3. To be an observational analysis (cross-sectional, cohort or case-controls).
4. To present results of associations based on linear regression analyses or Receiver Operating Characteristics Curve (ROC Curve) (for cross-sectional studies).
5. To be written in Portuguese, English and Spanish.

**Figure 1:**
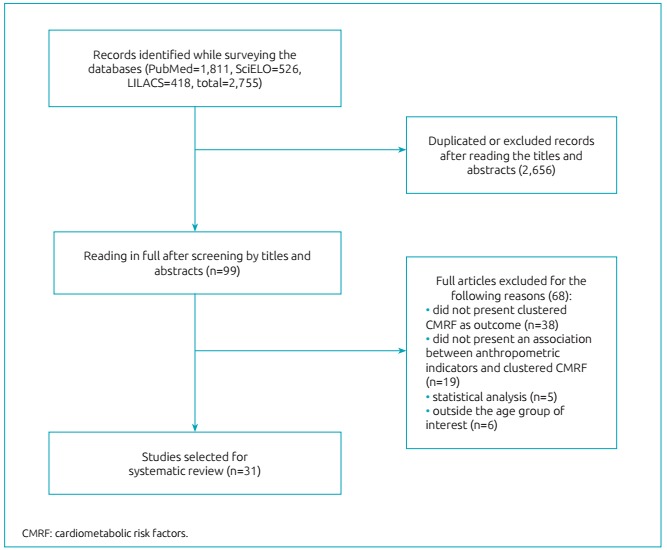
Flowchart of the process of selecting articles for the systematic review.

The review did not include studies with specific groups of patients with obesity or other conditions. The stages of paper selection can be observed in Figure 1.

The information selected in the articles to compose this review focused on the following items:

1. Descriptive: study, year of publication, study location, study design, sample size, age group and sex.
2. Methodological: characteristics of exposure and outcome measurements and statistical analysis used.
3. Description of the main findings.

Both the metabolic syndrome (MS) and the other clustered risk factors were deemed CMRF throughout the article, except in the tables, in which they will be approached according to the names used in the articles.

## RESULTS

As presented in Figure 1, 2,755 records were found, being 1,811 in PubMed, 526 in SciELO and 418 in LILACS. After excluding the duplicated records and reading the titles and abstracts, 99 articles remained to be read in full. Based on the full reading of the articles, 68 were excluded for the following reasons: did not present clustered CMRF as outcome (n=38); did not present any association between anthropometric indicators and clustered CMRF (n=19); did not present results of associations based on linear regression analyses or ROC curve (for cross-sectional studies) (n=5); and did not report data on children and adolescents (n=6). At the end, 31 articles were selected for the systematic review.

### Data on location, design and study population

The evaluation included recent articles, published in the past five years (June 1^st^, 2011, until May 31^st^, 2016). Six papers were published in 2015; 13, in 2014; 6, in 2013; 2, in 2012; and 4, in 2011. Of the 31 studies analyzed, 18 were conducted in countries from the American continent, 6 from Europe, 5 from Asia and 2 from Africa. Most studies were cross-sectional, and only 6 were cohort analyses. Regarding the study population, in 26 of them participants were aged between 6 and 18 years old, and only 5 comprised subjects aged between 6 and 20 years. The sample size of the studies ranged from 65^11^ to 16,914^12^ participants. Two studies reported findings on the association of anthropometric indicators and clustered CMRF only for female participants^13^ ^,^ ^14^ (Table 1).

**Table 1:**
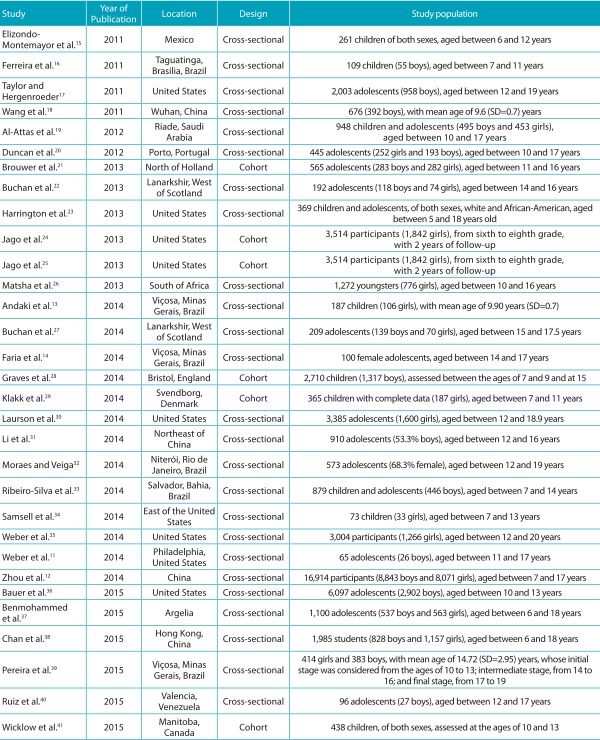
Characteristics of the studies included in the systematic review in relation to year of publication, location, methodological design and population.

SD: standard deviation.

### Data on exposure, outcome and statistical analysis

Concerning anthropometric measurements, 28 studies analyzed the ability of BMI as a predictor of clustered CMRF; 20, of WP; 10, of WHtR; and only 1 of the triceps, biceps, suprailiac and subscapular SF. Of the 31 studies, 9 compared the ability of BMI, WP and BD. However, of the nine studies that investigated BMI, WP and WHtR, only five presented a statistical test to verify the difference in the association between the three measurements. Of the eight analyses that investigated the predictor ability of BMI and WP, only two presented results referring to the statistical comparison between both measurements. The study comparing the ability of BMI and WHtR presented a result of the difference between both measurements, whereas the study that analyzed BMI, WP and SF did not. The outcome measurement mostly used by the studies was the MS (n=16); the other studies used different criteria to define clustered CMRF. Concerning statistical analysis, 19 studies used the ROC curve, 10 used linear regression, and 2 used logistic regression (Table 2).

**Table 2:**
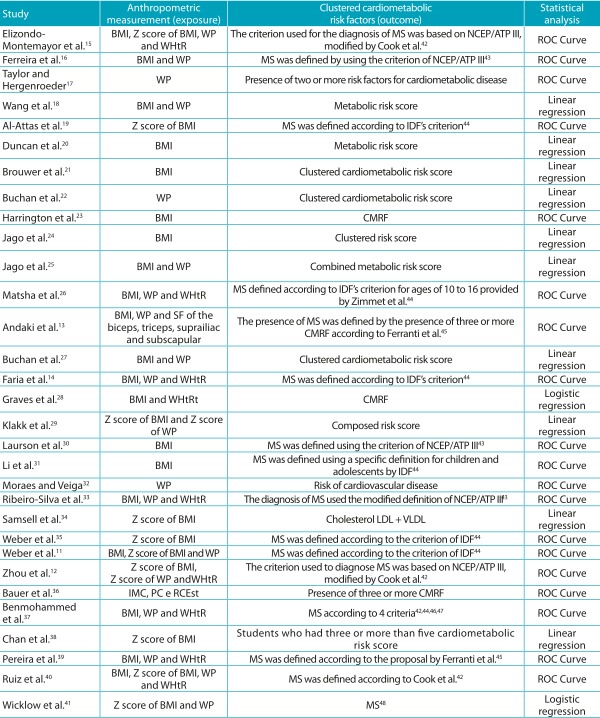
Characteristics of the studies included in the systematic review regarding the measurement of exposure, outcome and statistical analysis.

BMI: body mass index; WP: waist perimeter; WHtR: Weight-height ratio; SF: skinfolds; MS: metabolic syndrome; CMRF: cardiometabolic risk factors; IDF: *International Diabetes Federation;* NCEP: *National Cholesterol Education Program;* ATP: *Adult Treatment Panel*; ROC: *receiver operating characteristic*; LDL: *low-density lipoprotein*; VLDL: *very low-density lipoprotein*.

### Main findings

#### Cross-sectional studies

Of the 25 cross-sectional studies, only 3 did not show any association between some of the anthropometric indicators and MS or clustered CMRF.^11^ ^,^ ^20^ ^,^ ^27^ Six studies used linear regression for analysis. According to 3 of these studies, BMI explained the clustered CMRF from 2.4 to 35.0%.^20^ ^,^ ^34^ ^,^ ^38^ Only the study by Buchan et al.^27^ did not show any significant association between WP and clustered CMRF (β=0.050, *p*=0.118), and in the study by Duncan et al.^20^ the BMI was not able to predict clustered CMRF in boys (*p*>0.05). In the other studies, there was a positive and significant association of BMI and WP with clustered CMRF.^18^ ^,^ ^22^ WHtR was not investigated by any of these studies (Table 3).

**Table 3:**
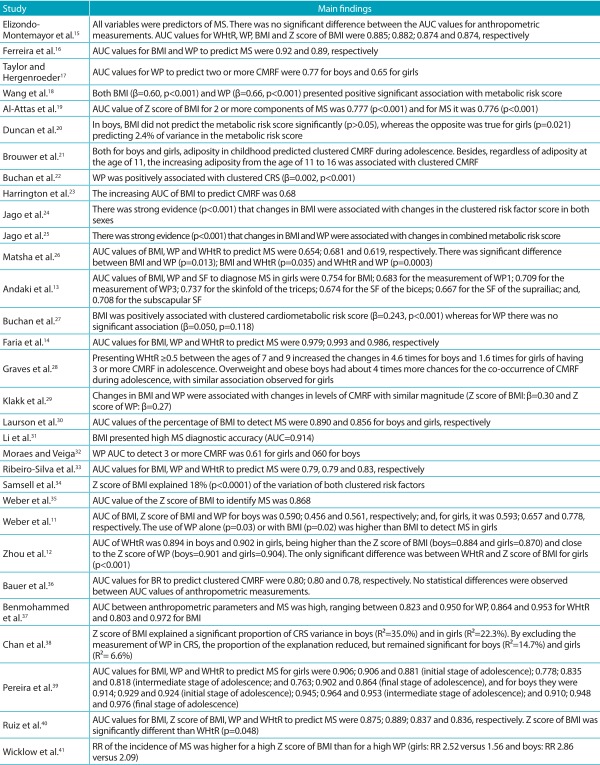
Main findings of the studies included in the systematic review.

BMI: body mass index; WP: waist perimeter; WHtR: Weight-Height ratio; SF: skinfolds; MS: metabolic syndrome; CMRF: cardiometabolic risk factors; AUC: accuracy; CRS: cardiometabolic risk score; RR: relative risk.

In the studies that used the ROC curve for analysis (n=19), the extension of AUC values for BMI was of 0.590 to 0.979; for WP, it was 0.561 to 0.993; and for WHtR was 0.619 to 0.986. Most studies found AUC higher than 0.700, regardless of the analyzed anthropometric measurement. In the study that analyzed the triceps, biceps, suprailiac and subscapular skinfolds, besides BMI and WP, as predictors of clustered CMRF, the extension of AUC values was of 0.667 to 0.737.^13^ According to the studies that compared the predictive value of BMI, WP and WHtR with the clustered groups, WHtR was higher than the Z score of BMI for girls (*p*<0.001);^12^ on the other hand, according to Ruiz et al.,^40^ the Z score of BMI was higher than WHtR (*p*=0.048). The studies by Elizondo-Montemayor et al.^15^ and Bauer et al.^36^ showed no statistical difference between anthropometric indicators to predict clustered CMRF. However, the study by Matsha et al.^26^ showed significant difference, and WP was higher than BMI (*p*=0.013) and WHtR (*p*=0.0003), and BMI was higher than WHtR (*p*=0.035). In the study that presented the comparison of the prediction of BMI and WP, the use of WP alone (*p*=0.03) or with BMI (*p*=0.02) was higher than the BMI to detect MS in girls^11^ (Table 3).

#### Longitudinal studies

Of the six cohort studies, four used linear regression and one used logistic regression for statistical analysis. Two studies verified the predictive power of BMI for clustered CMRF; three evaluated BMI and WP; and one analyzed BMI and WHtR. According to the findings in this study, there is evidence that BMI is a predictor of clustered CMRF.^21^ ^,^ ^24^ Changes in BMI and WP were associated with changes in levels of clustered CMRF (*p*<0.001).^25^ ^,^ ^29^ Still, according to Wicklow et al.,^41^ the relative risk of MS incidence was higher for a high Z score of BMI than for a high WP, both in girls and boys. In the single study that analyzed WHtR, the findings showed that the value of WHtR≥0.5 in childhood increased the chances of having three or more clustered CMRF in adolescence, and that being overweight and obese increased in up to four times the chances of co-occurrence of risk factors during adolescence for boys, with similar association observed for girls^28^ (Table 3).

## DISCUSSION

This systematic review was conducted with 31 studies that presented data regarding the association between anthropometric measurements and clustered CMRF in children and adolescents. Most studies were cross-sectional, and only six were cohort analyses. BMI was the most investigated anthropometric measurement, present in 28 studies; and SF was the least investigated measurement - included in only one study. MS was used by most studies as an outcome measurement. According to the cross-sectional studies, anthropometric measurements were associated with clustered CMRF both in boys and girls. According to the findings in longitudinal analyses, changes in adiposity in childhood predict changes in levels of clustered CMRF in adolescence.

Regarding methodological criteria, it was possible to observe there was no consensus between the studies to define the outcome variable. The most used outcome measurement in the studies was MS (16 analyses); however, seven different criteria were used for its definition. This was also observed among studies that clustered the CMRF: some considered the presence of two or more risk factors as a cluster, whereas others considered the minimum of three factors. The names used in the studies also varied, for example: “metabolic risk score”, “combined risk score”, among others. The methodological differences between the criteria used to define the outcome measurement make it difficult to compare the studies, and, consequently, prevent the inference of power of the anthropometric measurements in the prediction of risk factors.

Of the 31 studies analyzed, only 3 (all cross-sectional) did not observe any association between anthropometric indicators and the clustered CMRF.^11^ ^,^ ^20^ ^,^ ^27^ Generally, among the cross-sectional studies, there was significant positive association of BMI, WP and WHtR with the clustered CMRF. Of the 25 cross-sectional studies, 19 used the ROC curve as statistical analysis, and AUC as a measurement to express the outcomes. AUC is a usual summary measurement for the performance of a test (i.e., anthropometric indicators) to discriminate a specific outcome (i.e., clustered CMRF). When it comes to the AUC value, the closer to 1, the highest the ability of the test to discriminate the outcome; therefore, values with extension from 0.70-0.79 can be considered good; from 0.80-0.89, very good; and from 0.90-1.00, excellent.^49^ ^,^ ^50^ Most studies analyzed in this review found AUC higher than 0.7, regardless of the anthropometric measurement analyzed. According to the longitudinal studies, having increased values of BMI, WP and/or WHtR in childhood increases the chances of having clustered CMRF in adolescence.

The findings in the studies that compared the predictive power of anthropometric measurements with clustered CMRF were diverging. In one of the analyses, WHtR was higher to the Z score for BMI in girls,^12^ whereas in two other studies the BMI was higher than the WHtR.^26^ ^,^ ^40^ Still, two other analyses showed no statistical difference between anthropometric indicators to predict the clustered CMRF.^15^ ^,^ ^36^ Regarding WP, a study found the superiority of this measurement in relation to WHtR^26^, and two studies found it in relation to BMI.^11^ ^,^ ^26^ Besides, in the study by Weber et al.^11^, the use of WP alone or with BMI was higher to BMI to detect MS in girls.

The decision about which measurement to use to predict clustered CMRF was the target of several previous publications and reviews.^4^ ^,^ ^5^ ^,^ ^6^ In the systematic review by Reilly et al.,^6^ nine studies compared the ability of BMI versus WP in the diagnosis of CMRF in children and adolescents, and three presented two or more CMRF as outcomes. The findings showed that the AUC of both measurements in the diagnosis of CMRF was similar. In this review, according to two cohort studies, the magnitude of the associations of BMI and WP in the prediction of clustered CMRF was also similar,^25^ ^,^ ^29^ whereas in the study by Wicklow et al.^41^, also with a cohort design, the relative risk of MS incidence was higher when the Z score of BMI was high in relation to WP, both in boys and in girls. On the other hand, WP was higher in relation to BMI in two other analyses.^11^ ^,^ ^26^ However, both studies were cross-sectional, and one of them included a sample of only 65 adolescentes,^11^ and this fact may decrease the force of evidence of the findings.

In the past years, WHtR has been suggested by some authors as the best measurement to predict risk factors in children and adolescents, to the detriment of BMI and WP.^3^ ^,^ ^5^ According to the studies that defend this idea, the fact of not presenting a measurement unit, correcting WP with height and having the possibility of presenting a single cutoff point for children and adolescents of both sexes make it more attractive than other indicators.^3^ ^,^ ^5^ In this review, out of the ten studies comparing the power of WHtR with BMI and/or WP for the prediction of clustered CMRF, only one found this indicator to be superior in relation to BMI and WP for females.^12^ In a systematic review conducted by Browning et al.,^5^ according to 13 cross-sectional studies with children and adolescents, WHtR and WP were more strongly associated with isolated CMRF than BMI. According to the authors, WHtR can be a more useful global clinical screening tool than WP and BMI, supporting the public health message: “keep your waist perimeter in less than half of your height”.^5^

Besides BMI, WP and WHtR, SF has also been investigated to predict CMRF in the pediatric population.^7^ ^,^ ^51^ ^,^ ^52^ According to Ali et al.,^7^ the accumulation of subcutaneous adiposity is a strong predictor of resistance to insulin and hypertriglyceridemia, and a stronger predictor of CMRF than visceral fat in children and adolescents. In the studies by Misra et al.,^51^ ^,^ ^52^ the SF of triceps and suprailiac were more strongly associated with the concentration of insulin at fasting, and subscapular SF presented higher AUC in relation to BMI to predict clustered CMRF in male adolescents, and higher than WP in female adolescents. However, in this review, only one study investigated the power of SF to predict clustered CMRF. According to the findings in this study, the SK was associated with MS with AUC values similar to BMI and WP.^13^ SK may present inter and intra-observer error that is higher than weight, height and WP measurements. Besides, in epidemiological studies, it is essential to involve trained and experienced evaluators, and these facts may make SF less attractive than other anthropometric indicators.^53^

This systematic review investigated the power of BMI, WP, WHtR and and SF as predictors of clustered CMRF in children and adolescents. A limitation of this study was the definition of the search in the last five years, and that may have prevented the inclusion of some articles. However, 31 studies were analyzed, and the number of articles included decreased according to the year of publication. Another limitation was the fact that the quality of the manuscripts was not assessed. Many of the studies included had small samples, and many of them showed results divided by sex and/or age group, thus considerably reducing the sample size in each analysis. The small size of the samples may have compromised the power of association of the anthropometric indicators and the ability of the studies to identify differences between the indicators to predict clustered CMRF.

According to the analysis of the articles included in this review, some knowledge gaps can be related, such as:

1. Lack of consensus for the cluster of CMRF, which makes it difficult to compare the findings between studies, as well as limits the inference on the theme.
2. Lack of studies investigating the power of WHtR and SK as predictors of clustered CMRF in childhood and adolescence.
3. Lack of studies comparing other anthropometric indicators, besides BMI and WP, as well as presenting statistical analysis of comparison.
4. Lack of cohort analyses investigating the ability of anthropometric indicators in the prediction of clustered CMRF.

The development of further studies considering these gaps can be relevant for the advance of knowledge in the field.

Based on the findings of this review, it is possible to infer that BMI, WP and WHtR were predictors of clustered CMRF in childhood and adolescence, presenting similar ability to predict these outcomes. These findings suggest that anthropometric indicators may represent an interesting tool for the epidemiological screening of clustered CMRF at early ages. Body weight, height and WP are simple, easy to get, low-cost measurements that could be institutionally assessed in the routine practice of several sectors (i.e., schools and family health units), as part of the health follow-up in the pediatric population.
